# Vasovagal Syncope during Office Hysteroscopy—A Frequently Overlooked Unpleasant Complication

**DOI:** 10.3390/medicina58111626

**Published:** 2022-11-11

**Authors:** Suset Rodriguez, Sergio Haimovich, Salvatore Giovanni Vitale, Luis Alonso, Jose Carugno

**Affiliations:** 1 Minimally Invasive Gynecology Unit, Obstetrics, Gynecology and Reproductive Sciences Department, Miller School of Medicine, University of Miami, Miami, FL 33136, USA; 2 Hillel Yaffe Medical Center, Technion-Israel Technology Institute, Hadera 32000, Israel; 3 Obstetrics and Gynecology Unit, Department of General Surgery and Medical Surgical Specialties, University of Catania, 95121 Catania, Italy; 4 Centro Gutenberg, Endoscopy Unit, 29012 Malaga, Spain

**Keywords:** hysteroscopy, office hysteroscopy, vasovagal reaction, vasovagal syncope, reflex syncope, neurocardiogenic syncope

## Abstract

Due to technological advancements in miniaturization of instruments and improved optics, the number of office hysteroscopic procedures has increased over time. Office hysteroscopy is preferred due to avoidance of general anesthesia and decreased overall cost. Vasovagal syncope has been implied as the most common complication. Vasovagal syncope is associated with inappropriate reflex vasodilation and bradycardia in the setting of an acute malfunction between the autonomic nervous system and the cardiovascular system; however, there is no mortality associated with vasovagal syncope. A management strategy for acute vasovagal reflex during office hysteroscopy is proposed in order to manage this common complication.

## 1. Introduction

With the advancement of surgical technology and the progress in minimally invasive surgical access, outpatient and in-office procedures now have an important role in modern gynecology. The volume of ambulatory surgery alone has seen an increase in 16% from 1994 to 2014 [1]. Although it is difficult to appropriately measure the amount of outpatient procedures performed, it has been estimated that 10–12% of ambulatory procedures are performed in an office setting [2]. Hysteroscopy is considered the gold-standard technique for the evaluation and treatment of patients with intrauterine pathology [3]. Due to technological advances in miniaturization of instruments and improved optics, the number of office hysteroscopic procedures has increased over time [4]. The office setting is frequently considered the preferred location for hysteroscopic procedures, reducing the risks of anesthesia and lowering the overall cost of the procedure. The main risks associated with office hysteroscopic procedures generally involve pain, infection, bleeding, and vasovagal reaction [5].

## 2. Purpose

Vasovagal reaction has been implied to be the most common complication; however, the risk of a vasovagal reaction is low, ranging from 0.21–1.85% for office hysteroscopy [6,7,8,9,10]. The clinician performing in-office hysteroscopy must be familiar with the pathophysiology of this condition to quickly identify and appropriately manage it when encountered. In this narrative review, we present a comprehensive review that will guide the gynecologist performing office hysteroscopy in the evaluation and management of a patient with vasovagal presyncope and syncope.

## 3. Pathophysiology

Vasovagal syncope (VSS) is the most common type of reflex syncope [11]. Vasovagal syncope has been defined by the 2015 Heart Rhythm Society expert consensus statement as syncope that usually (a) occurs with an upright posture of more than 30 s or with exposure to pain, emotional stress, or medical settings; (b) is associated with diaphoresis, warmth, pallor, and nausea; (c) is associated with hypotension and relative bradycardia; and (d) is followed by fatigue [12]. Furthermore, presyncope is defined as the symptoms before the syncope, i.e., lightheadedness, visual disturbances, and altered level of consciousness without complete loss of consciousness [9]. Vasovagal syncope is associated with inappropriate reflex vasodilation and bradycardia in the setting of an acute malfunction between the autonomic nervous system and the cardiovascular system [13]. In most cases, VSS is not associated with cardiovascular, neurological, or any other underlying disease [14] and patients suffering from VSS are generally normotensive with appropriate blood pressure (BP) control otherwise [15]. It is estimated that approximately 50% of individuals experience a VSS episode at least once in their lifetime, and most individuals will only experience one episode [15].

Vasovagal syncope occurs when there is a sudden increase in vagally-mediated parasympathetic tone which leads to a decrease in heart rate or withdrawal of parasympathetic tone, or a combination of both. This then causes a decrease in systemic vascular resistance and preload, resulting in low blood pressure, sometimes associated with bradycardia. The result is a decrease in mean arterial blood pressure below what is required to maintain adequate cerebral perfusion, which subsequently results in the syncopal episode [12]. Specifically, during hysteroscopy, the parasympathetic nervous system can be activated by the handling of the cervix or uterine cavity [5].

Another commonly used model is the Bezold–Jarisch reflex. It begins by excessive venous pooling, which decreases volume in ventricles, and results in increased inotropy of the ventricles. Left ventricular sensory receptors are activated due to increased inotropy, which then increase vagal output to the Central Nervous System (CNS), ultimately increasing parasympathetic output and decreasing sympathetic output. With increased parasympathetic output, the result is vasodilation associated with bradycardia [16], and thereby cerebral hypoperfusion and syncope occurs [17].

When syncope is associated with anxiety, emotional triggers, or pain, the pathway is postulated to be via direct action at the medulla, which triggers parasympathetic efferent output resulting in hypotension and bradycardia.

Overall the pathophysiology is rather complex as there are different clinical presentations, distinct outcomes, and various drugs that can induce the resulting hypotension and bradycardia such as isoproterenol, nitroglycerin, and clomipramine [18].

## 4. Risk Factors

It is important to identify risk factors for VSS in patients presenting for office hysteroscopy. Specific data regarding vasovagal reactions with hysteroscopy are limited, however, Agostini et al. have demonstrated higher a rate of vasovagal reaction with rigid hysteroscopes compared to flexible hysteroscopes as well as with the use of CO_2_ as distending media compared to saline solution [19]. On the other hand, the use of the vaginoscopic technique and topical endometrial anesthesia also been shown to decrease the risk of vasovagal syndrome in office hysteroscopy [8,20].

Based on data from the Heart Rhythm Society consensus statement, vasovagal syncope is seen more commonly in females of 17 years old on average [12]. Furthermore, prior syncopal episodes, history of bronchial asthma, and female gender have been identified as predictors for syncope recurrence, as noted in a systematic review by Aydin et al. [18].

The majority of studies that have identified risk factors for vasovagal reactions and syncope are from procedures other than hysteroscopy. In patients presenting for blood donation, identified risk factors include young age, female, white, and low BMI/weight amongst other factors. Unobservable characteristics include low blood pressure, elevated pulse, history of vasovagal reaction, greater anxiety, pain, sleep duration less than 8 h, more than 4 h since last eating, and first time donating blood [21,22,23,24]. Similar findings were shown when studying patients undergoing ambulatory pain procedures, which found that first time undergoing procedure, low systolic blood pressure pre-procedure and first time undergoing the procedure were risk factors for VSS [25,26]. Family history of VSS has also been noted as a risk factor [27].

## 5. Diagnosis

Diagnosis of vasovagal syncope is made clinically based on detailed history, physical exam, and witness by bystander if possible. Several risk scores have been proposed to aid in the diagnosis of syncope [28]; however, traditionally history and physical examination suffice. It is typically preceded by prodromal symptoms and/or identifiable triggers [11]. Strong emotions, sudden pain, medical environments, and events that activate the parasympathetic reflex such as micturition have also been identified as triggers for VSS [12,29]. Prodrome symptoms associated with vasovagal reaction are warmth, diaphoresis, nausea, epigastric discomfort, abdominal cramps, weakness, lightheadedness, yawning, hyperventilation, impaired hearing, desire to sit down or to leave the room, and pallor [16,30,31]. It is common for older adults to not experience prodromal symptoms [31].

Clinical signs then proceed to facial pallor which results from decreased blood flow as a consequence of low blood pressure and sympathetic vasoconstriction [32]. Other signs are hearing loss, difficulty concentrating, losing awareness of surroundings then finally falling down with loss of consciousness. Other associated findings may include sinus tachycardia prior to syncopal episode, with subsequent decrease in heart rate [33]. Duration of unconsciousness has been described to last from 10–120 s [34].

Recommended diagnostic workup for all patients with a syncopal episode is an electrocardiogram (ECG) in order to assess for cardiac etiology, such as arrhythmias or ischemia, pulmonary embolus or hypertrophic cardiomyopathy [35]. The remainder of the diagnostic testing is ordered as clinically indicated based on history and physical exam [36] and is outside of the scope of this review.

## 6. Acute Management

**Figure 1:**
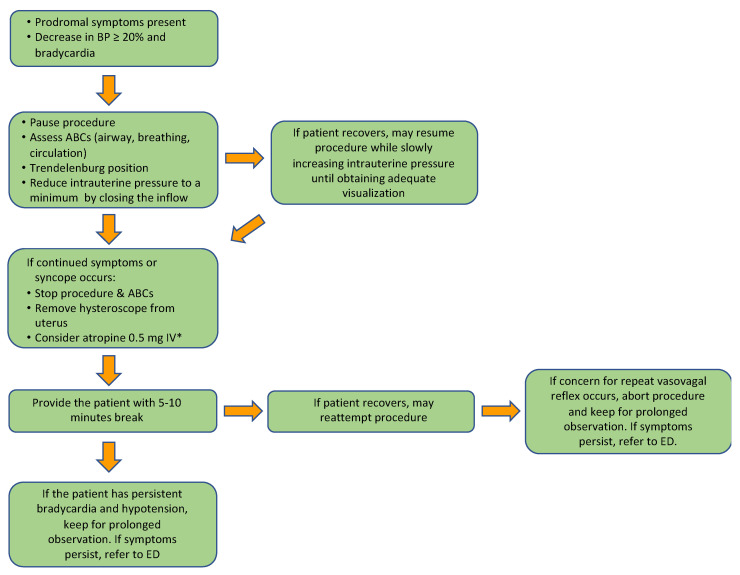
Proposed algorithm for acute management of vasovagal reaction and syncope during office hysteroscopy. * Dosing of atropine: 0.5 mg IV every 3–5 min, with maximum dose of 3 mg.

As vasovagal response is the most common complication during office hysteroscopy, it is important for the physician to be prepared with its management. Given the typically self-limited nature [37] and the rarity of the event, scant data exists on the acute management of vasovagal syncope during office hysteroscopy. In general, given that VSS is generally benign, medical treatment is only necessary if conservative measures fail [11]. Here we propose a strategy for acute management of vasovagal syncope during office hysteroscopy, Figure 1.

The immediate first step when a patient complains of prodromal symptoms (i.e., warmth, nausea, etc.) and vital signs demonstrate a decrease in blood pressure and/or bradycardia, is to pause the procedure and assess airway, breathing, and circulation (ABCs). The patient should then be placed in the Trendelenburg position in order to create a physical counter-maneuver thereby increasing central blood volume and cardiac output [20,38]. Changing the patient’s position to provide physical counter pressure was the only intervention noted to prevent vasovagal syncope when presyncope occurred in a recent systematic review [39]. At this time, we further recommend reducing intrauterine pressure to a minimum by closing the inflow; although there is no evidence to support this maneuver, it may minimize pain that is caused from uterine cavity distension thereby eliminating a potential trigger for vasovagal response [40,41]. At this point, if the patient has recovered, the procedure may be resumed while slowly increasing the intrauterine pressure until adequate visualization is obtained.

If continued symptoms persist or reoccur once continuing the procedure, then stop the procedure and remove the hysteroscope from the uterus while assessing ABCs. If bradycardia or symptoms persist, can consider administering atropine 0.5 mg intravenous (IV) every 3–5 min, with maximum dose of 3 mg [5], then provide the patient with a break of 5–10 min.

At this time, if the patient has recovered, then the procedure can be reattempted. If the patient has not recovered, then keep for prolonged observation and refer to the emergency department (ED) if patient has persistent symptoms. If when restarting the procedure there is concern for vasovagal response or syncope recurs, then abort procedure and also keep for prolonged observation with ED referral if symptoms do not resolve.

A similar algorithm used by Radavansky et. al. for the management of vasovagal response during office rhinologic manipulation procedures—involving providing smelling salts, stopping procedure and providing patients with 30 min break—noted that almost all patient with vasovagal reactions during the procedures recovered after resting for 15 to 30 min and were then able to complete the procedure without further complications [6].

Further management of vasovagal syncope is outside of the scope of this discussion.

## 7. Risk Stratification–When to Send to Emergency Department?

**Figure 2:**
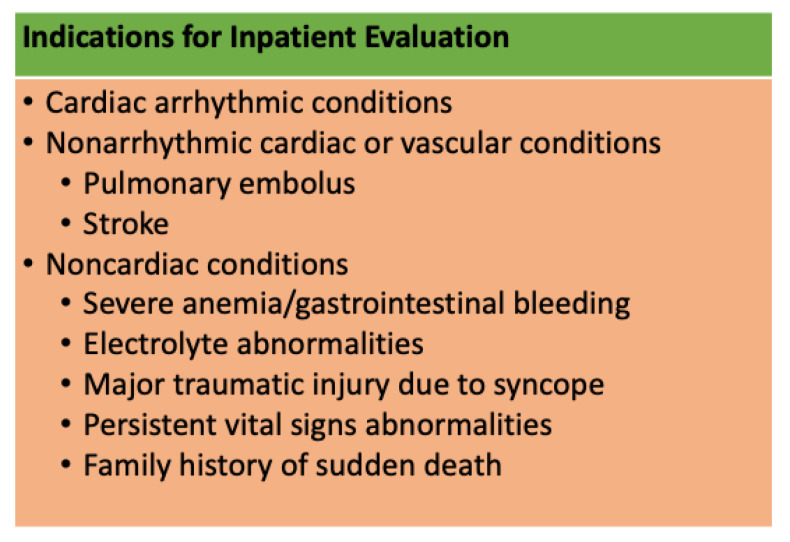
Indications for inpatient evaluation for patients with vasovagal syncope [11,36,42,43].

Patients with persistent vital sign abnormalities in the office require further evaluation in the ED. Evaluation in the ED consists of thorough history, physical exam, laboratory investigations, and a 12-lead ECG [11]. Further inpatient evaluation is recommended by The American Society of Cardiology/American Heart Association Task Force for patients suffering a syncopal episode with one or more conditions from the following three categories: cardiac arrhythmia, cardiac, or vascular nonarrhythmic conditions (pulmonary embolus, stroke), or noncardiac conditions (severe anemia/gastrointestinal bleeding, major traumatic injury due to syncope, or persistent vital sign abnormalities) [11,42]. Further proposed inpatient criteria include electrolyte derangements and family history of sudden death [36,43]. Figure 2 provides a summary of indications for inpatient evaluation. 

Further management of vasovagal syncope is outside of the scope of this discussion.

## 8. Severe Complications

No mortality has been associated with vasovagal syncope [44]. Overall, patients suffering from VSS have a high spontaneous remission rate; as such, long-term risk of death is similar in patients with vasovagal syncope compared to patients without syncope [45,46]. Hospital evaluation is unlikely to improve long-term outcomes, with the exception of patients with frequent episodes with associated injury risks [11].

## 9. Summary

Due to technological advancements with miniaturization of instruments and improved optics, the number of office hysteroscopic procedures has increased over time. Vasovagal syncope has been implied as the most common complication. Vasovagal syncope is associated with inappropriate reflex vasodilation and bradycardia in the setting of an acute malfunction between the autonomic nervous system and the cardiovascular system; however, there is no mortality associated with vasovagal syncope. A management strategy for acute vasovagal reflex during office hysteroscopy is proposed in order to manage this common complication during the procedure as well as risk stratification for emergency department evaluation and recommendation for admission.

## Data Availability

Not applicable.
